# Epidemiology, Virology, and Control of Highly Pathogenic Avian Influenza in Kazakhstan

**DOI:** 10.3390/pathogens14111084

**Published:** 2025-10-24

**Authors:** Zhandarbek Bekshin, Aliya Temirbekova, Zhadyrassyn Nurbekova, Nurgul Amirkhanova, Akbota Satenova, Albert Askarov, Kunsulu Zakarya, Yergali Abduraimov, Aralbek Rsaliyev

**Affiliations:** 1 Republican Collection of Microorganisms, Astana 010000, Kazakhstan; 2 Department of Biotechnology and Microbiology, L.N. Gumilyov Eurasian National University, Astana 010000, Kazakhstan; 3 National Holding Qazbiopharm, Astana 010000, Kazakhstana.rsaliyev@qbp-holding.kz (A.R.)

**Keywords:** avian influenza (AIV), wild birds, poultry, H5N1, H5N8, Kazakhstan, outbreaks, migration routes, epidemiology, control strategies

## Abstract

This review analyzed the epidemiology and impact of HPAI (H5N1 and H5N8) in migratory birds and poultry in Kazakhstan, with a special focus on outbreaks from 2005 to 2024. A comprehensive bibliographic analysis of publications on avian influenza in Kazakhstan over the past 20 years was performed. This review mainly included epidemiological studies of virus detections in wild and poultry in Kazakhstan. Migratory wild birds, in particular, played a key role in the spread of the virus. An analysis of the migration chronology of HPAIV-carrying birds prior to their entry into Kazakhstan was conducted, as well as a comparative analysis of seasonal and water landscape characteristics in previous habitats. The complexity and risks associated with the virus, together with the limited number of current scientific studies in Kazakhstan, require further research to reduce its impact on animals and the ecosystem as a whole.

## 1. Introduction

Highly pathogenic avian influenza (HPAI) is a form of bird flu caused by certain strains of influenza A virus, primarily subtypes H5 and H7, which cause severe disease with high mortality in poultry and can rarely be transmitted to humans, causing severe disease and even death [1]. Bird flu (avian influenza) has been known since the early 20th century, but modern highly pathogenic strains (H5N1and H5N8) emerged in the late 1990s in Southeast Asia. Documented cases of the virus crossing the species barrier, H5N1 into humans, were first recorded in 1997 in Hong Kong; since then, more than 900 cases of infection have been identified in 25 countries, with a mortality rate of over 50% [2].

According to the WHO and the US Centers for Disease Control and Prevention (CDC), the avian influenza virus (influenza virus A, a single-stranded RNA virus) belongs to the *Orthomixoviridae* family, a classic example of a constantly evolving pathogen, with changes in its antigenic structure occurring due to two key mechanisms: antigenic drift and antigenic shift. Antigenic drift is a process in which minor changes are introduced into key viral epitopes through point mutations in the viral genome. The segmented nature of the influenza virus genome also allows it to undergo genetic reassortment, which can lead to a process known as antigenic shift, resulting in significant changes in the antigenicity of the virus [3]. During reassortment, both external (HA and NA) and internal (PB2, PB1, PA, NP, M, and NS) gene segments may be exchanged. Antigenic shift does not always result in a new subtype, but it can produce a virus with new biological and pandemic potential. Such changes may enhance virulence and tropism, increase transmission, and alter the host range [4,5].

Up to 50 million birds migrate through Kazakhstan annually, facilitating the introduction of viruses from Europe, Asia, and Africa [6]. Such contacts facilitate the exchange of avian influenza viruses between bird populations from Southeast Asia, Europe, and North Africa [7]. The leading role is played by the Central Asian Flyway (CAF), which covers most of the country. This route connects the nesting areas of waterfowl and near-water birds in Siberia and Central Asia with wintering grounds in South Asia, including the Indian subcontinent and along the Indian Ocean coast [8]. The northern water bodies of Kazakhstan are characterized by the presence of various species of wild birds migrating from Africa, Europe, and Central and Southeast Asia. Such interpopulation interactions can lead to genetic reassortment of influenza virus subtypes and the emergence of new strains [9,10]. This circumstance makes the country particularly vulnerable to the spread of highly pathogenic avian influenza virus (HPAIV).

Wild birds serve as a natural reservoir of the virus and are a key factor in the spread of bird flu. During flight, when stopping at water bodies, infected birds excrete the virus with excrement and respiratory secretions, which leads to infection of other birds. Detection of the bird flu virus in nesting birds emphasizes the stability of viral circulation in the ecosystem. Cases of infection of wild birds in Kazakhstan coincided with the seasonal migration of bird species such as *Anser* spp., *Anas crecca*, *Cygnus olor*, *Larus* spp., *Tringa* spp., *Phoenicopterus roseus*, *Anas platyrhynchos*, and *Ardea cinerea*. The listed species are of the greatest epidemiological significance, which require monitoring for early warning of outbreaks [6,11].

Poultry as an object of outbreaks entails economic losses and threats to humans [6,12,13,14]. Poultry can become infected with influenza through contact with wild birds if biosafety rules are not observed, sanitary barriers are absent, wild birds have free access to feed and water, different types of birds are kept together, and non-sterilized equipment and vehicles are used [15]. The main control for wild birds is environmental monitoring, ringing, and analysis of samples from migration sites, and for domestic birds, veterinary supervision, quarantine, vaccination, and farm reporting [16].

This review summarizes the epidemiological history of HPAI subtypes H5N1 and H5N8 in Kazakhstan based on the scientific literature and official agency reports, with the aim of evaluating implemented biosecurity measures, assessing the national control strategy, and identifying factors that may explain the absence of documented human transmission [2]. Furthermore, the limited data on the evolution and ecology of avian influenza viruses in the region underscores the need for systematic monitoring of the disease in migratory birds.

## 2. Bibliographic Analysis

### 2.1. Study Selection and PRISMA Flow Diagram

The search strategy yielded 762 records (Scopus, Web of Science (WoS), PubMed, and the Cochrane Library). The search was conducted in the listed databases on 7 July 2025 using the search string with the terms “avian influenza” and “Kazakhstan”, covering publications from 2005 to 2025. Record selection was carried out in two stages. At the first stage, publications were screened based on their titles and abstracts, excluding studies unrelated to the scope of this research. Only original research articles and review papers published in English were included. All records were double-checked by two independent researchers to remove duplicates. After removing 107 duplicates, 655 records were screened. Of these, 600 were excluded based on title and abstract. At the second stage, the full texts of the articles selected at the first stage were reviewed. The exclusion criteria were studies addressing other countries, viruses not related to avian influenza, human influenza, studies on other animal species, and conference abstracts. The eligibility criteria were defined a priori and included studies published between 2005 and 2025, conducted in Kazakhstan, involving avian influenza subtypes of low or high pathogenicity and restricted to the bird host. The starting point was selected because the first outbreaks of highly pathogenic avian influenza in Kazakhstan occurred in 2005. At this stage, articles that did not meet the previously established criteria were excluded. As previously reported, two researchers independently selected publications and filtered the information to improve methodological strength, and disagreements were resolved through public discussions with the research team.

**Figure 1:**
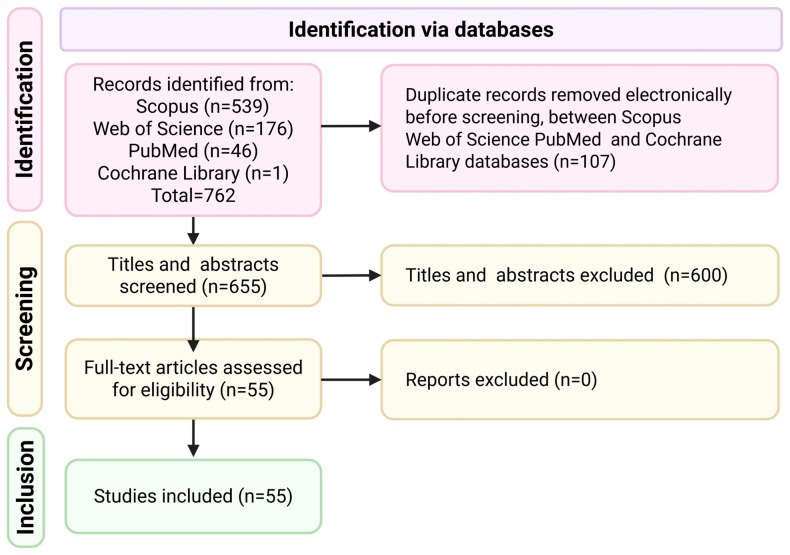
Prisma flow diagram of the literature search and selection process used in this study.

Finally, 55 articles met the inclusion criteria and were included in the bibliometric analysis. The study selection process is summarized in the PRISMA flow diagram (Figure 1). Of these, 37 records were common to the Scopus (6 individual records) and WoS (12 individual records) databases, and 1 record was common across all previously listed databases.

The final PRISMA flow diagram (Figure 1) illustrates all stages of the study selection: identification, duplicate removal, screening, exclusions, and inclusion.

### 2.2. Bibliometric Analysis

**Figure 2:**
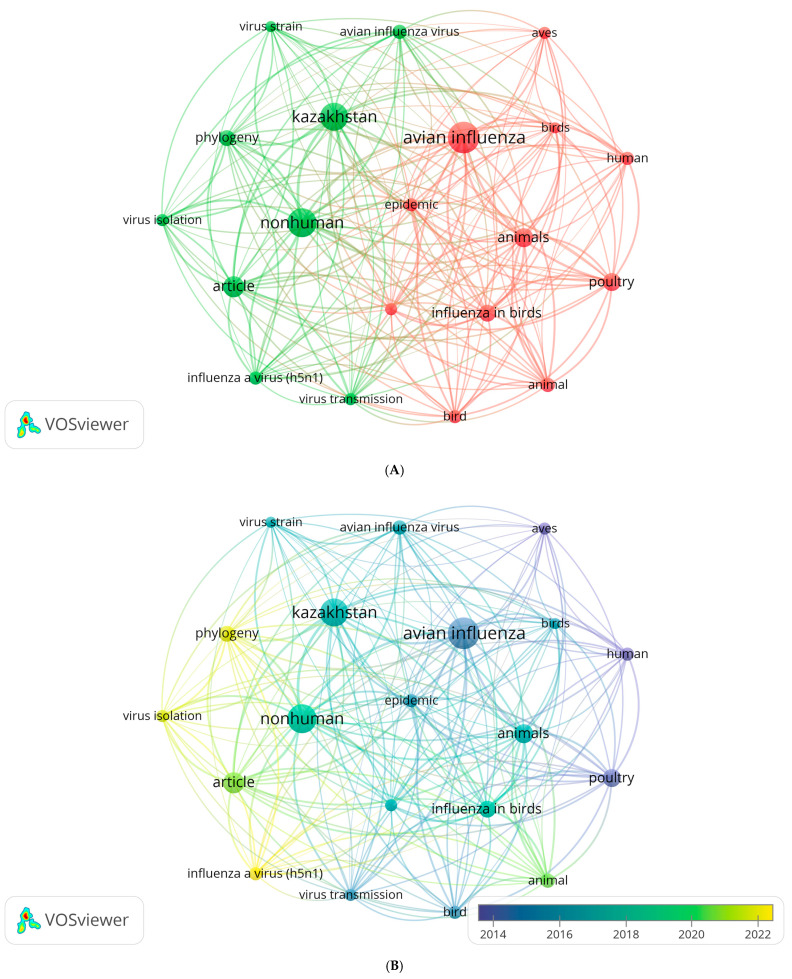
(**A**) Keyword co-occurrence network map based on 55 publications related to avian influenza in Kazakhstan (2005–2025). Node size indicates frequency of occurrence, edge thickness indicates co-occurrence strength, and colors represent thematic clusters. (**B**) Overlay visualization of the keywords used in avian influenza research in Kazakhstan. The color gradient reflects the temporal distribution of terms in the literature: blue indicates earlier topics, while yellow represents more recent ones. The size of the circles represents the frequency of appearance of the keywords.

Bibliometric indicators were analyzed using the VOSviewer, version 1.6.20 (https://www.vosviewer.com, accessed on 20 August 2025) (Figure 2). Co-occurrence network and overlay visualizations of keywords from the screened publications were generated to identify research trends and their temporal evolution. After merging keywords with the same meaning, 20 keywords with more than 10 occurrences were identified from these 55 publications.

The co-occurrence analysis of keywords revealed two distinct thematic clusters (Figure 2A). The red cluster reflects the epidemiological and epizootiological focus of studies, including terms such as “avian influenza”, “birds”, “poultry”, “animals”, and “human”, which are associated with outbreak investigations, host interactions, and public health implications. The strongest links were observed between “avian influenza”, “birds”, and “poultry”, reflecting research interest in the spread of infection among domestic and wild bird populations, the role of migratory species in viral transmission, and the resulting implications for the poultry industry and agriculture. The term “human” within this cluster indicates a link to public health issues and the risks of interspecies viral transmission to humans.

In contrast, the green cluster represents the molecular and phylogenetic aspects of research, encompassing keywords such as “Kazakhstan”, “virus strain”, “virus isolation”, “phylogeny”, and “influenza A virus (H5N1)”, indicating studies centered on virus characterization and strain evolution in the region. Studies within this cluster are aimed at identifying the evolutionary relationships of Kazakhstan isolates with viruses circulating in other parts of the Eurasian region.

Figure 2B reflects the temporal dynamics of research directions based on the co-occurrence analysis of keywords. The blue color (average publication year: 2014) indicates that the identified terms (“avian influenza”, “birds”, “poultry”, “animals”, and “human”) represent the early stage of research focused on outbreaks, host species, and zoonotic transmission pathways. These terms reflect the ecological and epidemiological emphasis of studies investigating viral circulation among domestic and wild birds and potential spillover to humans. During the earlier phase (2014–2016), studies predominantly focused on the ecological and epidemiological dimensions of avian influenza in Kazakhstan. In the intermediate period (2017–2020), research attention expanded toward molecular virology and regional epidemiology. The appearance of the term “Kazakhstan” in the center of the map indicates an increase in the number of local publications related to the analysis of outbreaks, the identification of strains, and the comparison of data with international databases. The connections between the terms “Kazakhstan” and “virus strain,” “phylogeny,” and “nonhuman” reflect the development of virus genetic typing and phylogenetic studies. This period witnessed a shift from descriptive epidemiological analysis to an analytical approach that included an assessment of genetic diversity and factors affecting viral adaptation to new host species. In contrast, terms marked in yellow (average publication year: 2022), such as «phylogeny», «virus isolation», and «influenza A virus H5N1 subtype», indicate a clear shift toward molecular and phylogenetic investigations aimed at understanding virus evolution and genetic diversity. The term “virus isolation” emphasizes the importance of experimental isolation of isolates for subsequent genomic analysis, while the term “phylogeny” reflects the emphasis on the evolutionary and phylogenetic relationships between H5N1 strains. The term “influenza A virus H5N1 subtype” is of particular importance, denoting a shift in the focus of research to highly pathogenic forms of the virus, which is associated with the need to assess their potential danger to agriculture and wildlife.

In this context, the increasing scientific contribution of Kazakhstan to both regional and global avian influenza research highlights its strategic importance in the surveillance, prevention, and control of highly pathogenic avian influenza viruses.

## 3. Epidemiology and Molecular Genetic Characterization of HPAI Viruses in Kazakhstan

**Figure 3:**
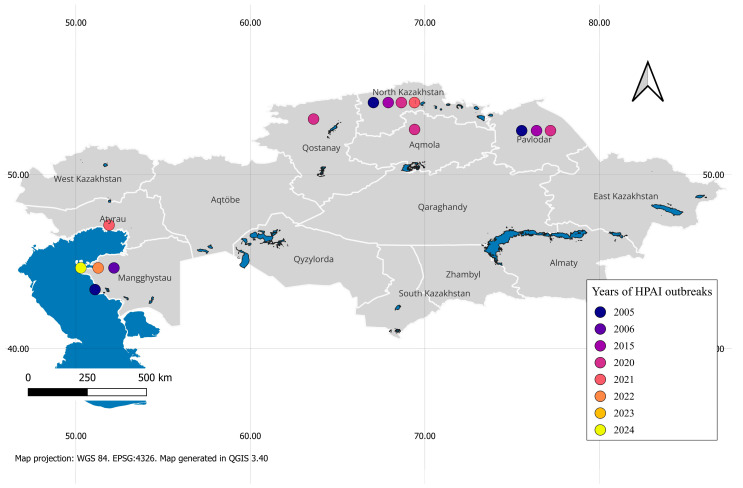
Map of HPAI distribution in Kazakhstan from 2005 to 2024. The map shows the years of HPAI outbreaks confirmed. On the map of Kazakhstan, white contour lines delineate the administrative units of the country, the regions, with their names indicated at the center of each. Outbreaks affected primarily the northern regions—North Kazakhstan, Pavlodar, Qostanay, and Aqmola—as well as the western part of the country, including Aktau and Atyrau. The map was created using QGIS version 3.40.3-Bratislava software (https://qgis.org, accessed on 20 August 2025).

Various strains of avian influenza viruses are detected in Kazakhstan, including HPAI, such as H5N1 and H5N8, which have repeatedly caused outbreaks in both wild and domestic birds. Outbreaks of HPAIV (H5N1) among wild birds were confirmed in 2005 and 2020–2021 in northern Kazakhstan, in 2006, 2021, and 2023–2024 in western Kazakhstan, and sporadically in 2015 and 2022. Among domestic poultry, outbreaks were in the northern regions, particularly in 2005 and in 2020–2021 [6,11,15,17]. Figure 3 demonstrates a map of the chronology of HPAI outbreaks by region of Kazakhstan, confirmed by studies of reference laboratories and research organizations, and reflected in publications of high-ranking journals and reports of international organizations.

In 2005, the first outbreaks of HPAI were recorded in four regions of Kazakhstan—Pavlodar, Akmola, North Kazakhstan, and Karaganda—among domestic birds. By the end of the same summer, the epizootic situation had become even more acute: the HPAI virus subtype H5N1, clade 2.2 (Qinghai-Siberian group), was detected in a sample of domestic goose in the Pavlodar region [18] in northern Kazakhstan [11,19]. According to the WOAH, by the beginning of August 2005, losses amounted to 600 geese that died naturally and 2350 individuals that were culled [20,21,22]. The presumed migration of birds indicates that the gene virus A/domestic goose/Pavlodar/1/05 (H5N1) (GS/1/05)—in domestic geese, which was detected in northern Kazakhstan in 2005—circulated among wild migratory birds along the route Qinghai → Russia (Siberia) → Kazakhstan → Europe flyway [23]. In 2005, bird flu was detected in Russia, Mongolia, Romania, Turkey, and Thailand [20]. The presence of European segments may be the result of secondary introduction from wild birds overwintering in Southern Europe. It was determined the complete genome sequence of the highly pathogenic avian influenza strain A/Domestic Goose/Pavlodar/1/05 (H5N1) [19]. Based on genetic analysis of pathogenicity determinants in HA, NA, and NS1, the GS/1/05 strain was classified as HPAI, exhibiting increased tropism for mammalian cells and resistance to the antiviral effects of interferons and tumor necrosis factor. However, existing studies have focused primarily on migratory spread and genetic analysis of strains, while deficiencies in poultry biosecurity and management systems have remained insufficiently addressed. This gap in analysis limits a holistic understanding of the multifactorial nature of the 2005 outbreak, where anthropogenic factors played no less a role than natural migratory routes. In the spring of 2006, several cases of HPAIV infection were again recorded in wild birds in the northern Caspian region. In particular, the A/swan/Mangystau/3/2006 (H5N1) virus was isolated from a dead swan on the southeastern coast of the Caspian Sea. The HA/NA genes and other markers indicated a mixture of Asian and European lineages, which suggests a migratory origin from the Black Sea region, the Sea of Marmara, and the Caspian Sea [24]. Although this strain is characterized by the absence of a polybasic cleavage site in the HA segment, it had high virulence (IVPI > 1.2), which, according to the WOAH criteria, defines it as HPAIV. In the spring of 2015, A/flamingo/Mangistau/6570/2015 (H5N1) was detected in flamingos in the Caspian Sea region. The introduction of the virus is associated with a migration route connecting West Africa–the Caspian coast–India [25].

In autumn 2020, 11 outbreaks occurred in Kazakhstan in the northern and central regions (Akmola, Almaty, Kostanay, and Pavlodar regions), including poultry. In Northern Kazakhstan, near the Russian border, a highly pathogenic H5N8 virus clade 2.3.4.4b was detected [26]. In Russia, that same autumn, there was a large-scale epizootic event, affecting more than 10 regions, caused by H5N8 subclade 2.3.4.4b [27]. The A/chicken/North Kazakhstan/184/2020 (H5N8) virus detected in chickens in Northern Kazakhstan was the result of complex reassortment, likely associated with migration routes through Siberia and the Caspian region [28,29,30,31]. It has been reported that BLASTn (version 2.17.0) analyses of the strain A/Chicken/North Kazakhstan/184/2020 (H5N8) showed significant genetic similarity across all eight gene segments to highly pathogenic H5 viruses isolated from domestic poultry in the Middle East and West Africa, where they had caused outbreaks [32]. Furthermore, the H5 HA cleavage-site motif was identical to that observed in European viruses from 2016 to 2017, including outbreaks in domestic poultry in the United Kingdom. Before this, seven outbreaks of H5N8 were reported in Europe between May and August 2020 (one in Bulgaria and six in Hungary) [33,34]. Earlier this year, the H5N8 subtype strain also caused a major outbreak in Saudi Arabia, which killed 22,000 birds, confirming active Asian circulation [35]. Following outbreaks in Kazakhstan, in China, the A/goose/China/1/2021 (H5N8) virus turned out to be a reassortant, similar in some genes to those circulating in Kazakhstan and Russia, and the remaining genes to the South Korean isolates of November–December 2020, and it also belonged to the 2.3.4.4b subclade [36]. Studies by Ali et al. further demonstrated the closeness of H5 isolates recovered from chickens and ducks in southern Egypt to H5N8 strains isolated in Kazakhstan in 2020 [37].

In the fall of 2021, five additional outbreaks of infection were registered in Kazakhstan in the Akmola, Aktobe, Pavlodar, and North Kazakhstan regions. Quarantine was introduced in the affected areas. Restrictions on the export and domestic sale of live poultry and poultry products subsequently led to higher prices for eggs and poultry meat [6].

Kydyrmanov et al. noted that in the summer of 2022, a mass mortality of Caspian terns (*Hydroprogne caspia*), gulls (*Ichthyaetus ichthyaetus*), and gulls (*Larus cachinnans*) was recorded on the northeastern coast of the Caspian Sea. The outbreak resulted in the death of more than 5000 gulls and terns. The A/Caspian tern/Atyrau/9184/2022 (H5N1) virus detected in terns on the northeastern coast of the Caspian Sea was genetically close to Russian isolates and was likely introduced from the north via bird migration along the coast [17,27].

In late 2023–early 2024, a new wave of HPAI outbreaks occurred in Mangystau during winter, characterized by mass swan mortality along the eastern coast of the Caspian Sea. A/Mute swan/Mangystau/9809/2023 (H5N1) was detected, a reassortant that probably formed from a mixture of viruses circulating in birds migrating between Siberia and North Africa. In 2024, isolates A/Cygnus cygnus/Karakol lake/01/2024 (H5N1) and A/Mute swan/Karakol lake/02/2024 (H5N1) were detected in whooper swans from Lake Karakol, genetically close to the Moscow and Omsk strains, indicating mixing during migrations of ducks from Northern Russia to the Caspian Sea [11]. Tabynov identified the isolated strain A/mute swan/Mangystau/1-S24R-2/2024 as an HPAI virus based on the presence of the polybasic cleavage site PLREKRRKRGLF in the HA gene. Based on phylogenetic analysis, the strain A/mute swan/Mangystau/1-S24R-2/2024 was assigned to clade 2.3.4.4b. According to phylogenetic analysis based on the HA gene, the A/mute swan/Mangystau/1-S24R-2/2024 strain was genetically closest to the A/mute_swan/Poland/MB008-1/2024 (H5N1) strain collected from a dead swan in January 2024. It was distantly related to several vaccine strains: approximately 90% to A/duck/Novosibirsk/Novosibirsk/02/05, 91% to A/duck/China/E319-2/03, and 92% to A/reassortant/IDCDC-RG43A (H5N8) [38]. Subsequently, a strain was detected in gulls in Iraq at Lake Dukan that closely clustered with HPAI strains (A/mute/Mangistau/1-S24R-2/2024 (H5N1) and A/Cygnus cygnus/Lake Karakol/01/2024 (H5N1)), with DNA identity of 99.38% and 98.82%, respectively, confirming its spread across different species and countries [39]. It has been reported that the isolated H5N1 viruses undergo complex reassortment during their long-distance dissemination along different avian migratory flyways [11]. During 2023–2024, outbreaks of H5N1, H5N6, and H7N9 continued in Asian countries, including China, India, and Vietnam [6,40]. A summary analysis of HPAIV detected in Kazakhstan and the expected routes of their circulation in the world is summarized in Table 1.

**Figure 4:**
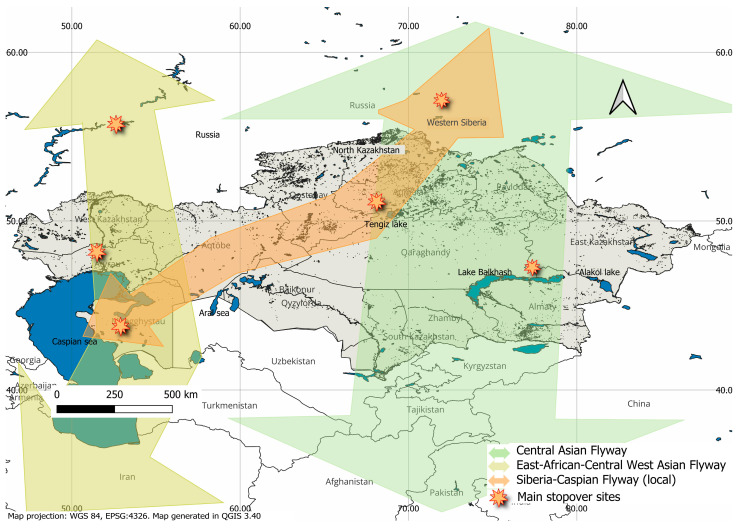
Bird flyways in Kazakhstan and their main stopover sites. The map shows two major global migratory routes and one local route crossing the territory of Kazakhstan. The two global flyways are the Central Asian route, which enters from Siberia (Russia) and continues southward toward Pakistan and India, and the East African–West Asian route, which enters from the Volga-Ural region. The main local route, the Siberia–Caspian route, extends from Siberia through Northern Kazakhstan to the Caspian Sea coast. The main stopover sites of migratory birds with epidemiological significance for the spread of HPAI are marked with yellow star symbols. The map was created using QGIS version 3.40.3-Bratislava software (https://qgis.org, accessed on 20 August 2025).

At the same time, Figure 4 reflects the main migratory routes of birds in Kazakhstan, which directly affect the spread of HPAI.

Furthermore, the main periods and places of distribution of highly pathogenic subtypes of the bird flu virus in Kazakhstan—H5N1 and H5N8—belonging to wild or domestic birds, are reflected in Table 2.

According to the data presented in the review of HPAI outbreaks, the Caspian Sea coast is the major stopover site for swans [49], and where spring, summer, and winter are the periods of circulation of these viruses, leading to mass outbreaks. It is swans, especially in winter on Lake Karakol, that are often the first to be hit by the virus, which indicates their high vulnerability. Whooper swans (*Cygnus cygnus*) in East Asia winter in China, South Korea, and Japan. Their breeding range extends from the wetlands of the Gobi Desert in the far west of Mongolia to the tundra rivers in the far east of Russia, as evidenced by ringing and satellite data. In recent years, whooper swans have attracted attention as an indicator species in connection with the spread of H5N1. H5N1 clades 2.2 and 2.3.2 were consistently detected in dead whooper swans from 2005 to 2010 in Mongolia [50]. Large populations of whooper swans, mute swans, mallards, and geese winter along the Caspian coast, which increases the risk of virus outbreaks. Waterfowl that frequent the coastal areas of the Caspian Sea and their habitats together represent a major potential global hotspot or high-risk region for the generation and transmission of highly pathogenic avian influenza viruses and other dangerous zoonotic diseases [51,52].

HPAI viruses in domestic poultry are genetically related to strains from the broad Eurasian wave of 2020–2021. Chicken genomes identified in Kazakhstan demonstrate clustering with neighboring regions of the Russian Federation and Europe [53]. Northern Kazakhstan is the main region where domestic poultry outbreaks are observed, mainly in autumn. The regions’ grain sector may contribute to risk, as harvest residues can attract wild birds that act as potential carriers of avian influenza, thereby increasing the likelihood of farm-level virus introduction.

Thus, having reviewed the outbreaks of HPAI among wild and domestic birds, several key aspects can be identified: the main region of Kazakhstan, the main objects, factors, and periods (Figure 2).

Table 3 reflects the virological features of HPAI detected in Kazakhstan, as well as their markers. Information on nucleotide sequence comparisons, including those of closest relatives, was obtained from the original articles, where the authors performed BLAST (version 2.17.0) alignments against the GenBank database. In our summary tables, we only list the closest relatives identified in the relevant studies. Detailed gene-segment attributions for all isolates are provided in Supplementary Table S1.

The analysis of the amino acid sequences of hemagglutinin from Kazakhstan isolates of the highly pathogenic avian influenza virus demonstrated a link between these strains with viruses from Siberia, the Middle East, and North Africa [55], confirming the role of bird migration routes in the spread of infection. In addition, genetic studies conducted in Kazakhstan in 2005 revealed the presence of key mutations, including E627K in the PB segment [41], associated with increased adaptation of the virus to mammals [56,57]. This mutation enhances polymerase activity at mammalian respiratory tract temperatures, while additional substitutions, such as D701N in PB2, have also been implicated in host adaptation [58]. By 2021, HPAI clade 2.3.4.4.b viruses began to predominate in Kazakhstan, as well as throughout the world, becoming a major global threat [59,60,61,62].

The review of HPAI strain studies reveals a shortage of comprehensive investigations that compare outbreaks across ecological conditions, the biodiversity of susceptible bird species, and the magnitude of mortality. Furthermore, collaborative research between field surveillance specialists and molecular researchers would enable a more rigorous assessment of the drivers and risks of HPAI spread.

## 4. Risk Factors for the Spread of HPAI in Kazakhstan

The main risk factors for the spread of HPAI in the world are wild bird migration routes, high poultry density, intensive farming, insufficient biosecurity on poultry farms, and climatic conditions that facilitate the spread of the virus [63,64,65]. In the national context, commercial output from industrial farms moves largely through regulated refrigerated supply chains, whereas a substantial backyard/household sector—especially in rural areas—relies on live-poultry markets and informal point-of-sale networks, increasing domestic–wild interfaces and movement-related transmission risk [11,15,66]. These live-bird marketing nodes are, therefore, identified as priority targets for targeted biosecurity and movement controls in national reports [66].

Kazakhstan is located in a high-risk zone due to the intersection of migration routes of birds connecting Asia, Europe, and Africa [39], including the Central Asian Flyway (CAF). Heat maps show that the highest risk of infection with avian influenza occurs in Northern and Southwestern Kazakhstan [25,38,43]. Avian influenza, especially its highly pathogenic forms (H5N1 and H5N8), remains one of the most significant transboundary threats in the field of veterinary science and zoonotic epidemiology [6].

Wetlands represent particularly high-risk stopover areas for migratory birds, especially in the grain-growing regions of northern Kazakhstan [67]. In Kazakhstan, the association between grain-growing areas and increased congregation of migratory waterfowl is considered a plausible hypothesis based on ecological observations and preliminary field surveys, though it requires further confirmation through targeted monitoring and quantitative studies. The epizootic of the H5N8 virus in poultry with parallel detection in wild birds confirms the vulnerability of this region in the years of unfavorable situations in Eurasia. The northeastern coast of the Caspian Sea serves as a transit point for millions of migratory birds [68], which is characterized by multiple introductions of the H5N1 virus [10]. Thus, in 2021, the H5N1 virus was isolated from flamingos on the coast of the Caspian Sea [13], and in 2022–2023, deaths of gulls and terns were recorded in the same region, indicating active virus circulation within a shared water basin [17]. These local outbreaks not only demonstrate the mechanisms of introduction but also pose a threat to the formation of permanent natural reservoirs of infection.

The risk of HPAI introduction and spread of the bird flu virus in Kazakhstan has a pronounced seasonal dynamic. In the Caspian Sea, H5N1 outbreaks are recorded mainly in winter and spring [6]. In the world, H5N1 outbreaks also show a clear seasonality with a high density of outbreaks in winter and early spring [69]. The seasonal cycle of H5N8 avian influenza circulation worldwide is lowest in summer and peaks in winter, with most outbreaks reported in the Northern Hemisphere, suggesting that the virus is more widespread during the cold season [70]. In northern Kazakhstan, however, a significant epidemiological risk was observed during the return migration of birds, when circulation of the H5N8 virus among poultry was documented mainly in the autumn months (September–October).

The greatest danger is posed by the northern and northeastern regions of the country, where the North Kazakhstan region, which occupies a leading place in terms of risk level, is characterized by a high density of wetlands (Kushmurun and Shaglyteniz lakes), poultry farming (up to 30% of the republican livestock), and the intersection of migration routes from Siberia to South Asia. The Akmola and Pavlodar regions, which also belong to the maximum-risk zone, have similar characteristics, but with a lower concentration of poultry farms. According to Chechet et.al., the virus is brought to poultry factories by synanthropic birds, humans, transport, feed, and other pathways [71,72].

An ecological assessment of the territory [6] showed that large agricultural zones of the country create conditions for the survival of the virus and cross-species transmission. Large pastures and free-ranging poultry, especially in the southern and western regions, contribute to an increased risk of contact between wild and domestic birds. Field observations confirm that transmission of the virus can occur within wild flocks without human involvement, making such outbreaks particularly difficult [73]. These features indicate the need to strengthen biosecurity measures both on farms and in wild areas.

With the intensity of circulation of avian influenza viruses and contacts between their different strains, the risk of the emergence of reassortments increases as a new challenge for epidemiological surveillance and vaccination. The phenomenon of reassortment emphasizes the need for active molecular genetic surveillance of influenza viruses circulating among agricultural and wild animals. This is especially important in regions with a high density of livestock and poultry farming, where the risk of coinfection and the emergence of new zoonotic variants is significantly higher [74]. It is suggested that the H5N1 genotype arose as a result of reassortment of H5N8 with LPAI as a result of phylogenetic and genotypic analysis conducted in China, where complete genome sequencing of 233 isolates was performed [53,75]. Particular concern is caused by the novel genetic reassortants of H5N1, detected in Kazakhstan in 2023–2024, which have resulted in mass mortality of swans [11]. These strains have been shown to be highly virulent and capable of spreading both in wild bird populations and among domestic birds, potentially posing a threat to humans as well [67,76]. According to previous studies, the sequence of the NP gene of the A/Cygnus cygnus/Karakol lake/01/2024 (H5N1) strains demonstrated 98.91% identity with the A/duck/Moscow/6131/2022 (A/H3N8) strain, and the NP gene of the A/Mute swan/Mangystau/9809/2023 (H5N1) strain had 99.19% identity with the A/duck/Moscow/6454/2023 (A/H11N9) strain [11]. These high sequence similarities suggest that the PB2, PB1, and NP segments of the Kazakhstan strains were likely derived through reassortment with low-pathogenic avian influenza viruses (LPAIVs), as supported by phylogenetic tree analyses presented earlier [11]. Functionally, these reassortants were linked to increased virulence in wild birds (mass swan mortality) and carry internal gene constellations from low-pathogenic lineages that may broaden the host range [11,77].

**Figure 5:**
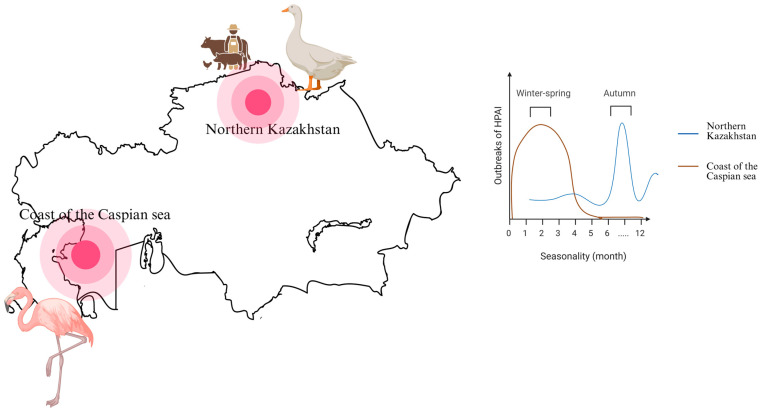
Geographic and seasonal features of the spread of HPAI in Kazakhstan. On the right side of the figure, the map of Kazakhstan shows two red circles indicating the main areas of HPAI occurrence—the coast of the Caspian Sea and northern Kazakhstan—which differ. The icon of a flamingo indicates the vulnerability to avian influenza among wild birds along the Caspian Sea coast, whereas the icon of a goose represents poultry infections in northern Kazakhstan. The symbol of a farmer with livestock denotes the high concentration of agricultural holdings in the northern part of the country. On the left side of the figure, the graph illustrates the seasonal peaks of avian influenza among birds, corresponding to these two regions. The scheme was created with Biorender.com (https://app.biorender.com, accessed on 20 August 2025).

At the same time, the detection of the low-pathogenic strain H9N2, which threatens poultry farming, underscores the risk of its potential mutation into more dangerous forms. These findings emphasize the importance of continuous molecular genetic surveillance of bird flu viruses in Kazakhstan for timely forecasting of epidemiological risks and development of preventive measures [32]. Kydyrmanov A., Karamendin K., and Daulbayeva K. investigated large-scale H5N1 epizootics in wildfowl, mass mortality events on the Caspian Sea, and global migration routes of the virus [17,25]. Karamendin K. and Kydyrmanov A. emphasized that the circulation of a rare H11 virus subtype—a reassortant between American and Eurasian lineages—indicates intercontinental exchange of viral gene segments along the Caspian Sea coast [78] (Figure 5).

Circulation of HPAI in populations of domestic and wild birds (Figure 5) is accompanied by risks of interspecies transmission of viruses to humans. Given the migration routes of migratory birds crossing the territory of Central Asian countries, including Russia, Uzbekistan, Kyrgyzstan, Tajikistan, and Turkmenistan, a transboundary zone of epizootic risk is formed [79,80].

Kazakhstan is involved in international trade and transport, including the movement of live poultry, feed, and poultry products, which increases the risk of HPAI importation from external territories [46]. Outbreaks of highly pathogenic avian influenza in neighboring countries, especially in China [36] and Russia [27], pose a potential threat due to close economic, logistical, and environmental ties.

Thus, considering the factors listed above, effective HPAI control in Kazakhstan requires an integrated approach that includes monitoring wild birds, farm biosecurity, restrictions on interregional movement of birds, and molecular surveillance of circulating strains to identify new dangerous variants (Figure 5).

The viruses were repeatedly introduced via bird migration routes, underwent reassortment and accumulation of characteristic mutations, and eventually shifted evolutionarily toward the globally dominant clade 2.3.4.4b.

## 5. Diagnostics of HPAI in the Field of Veterinary Medicine of the Republic of Kazakhstan

In recent years, laboratory tests have played a decisive role in confirming HPAI outbreaks. The most widely used methods are reverse transcriptase–polymerase chain reaction (RT-PCR) and its quantitative version, real-time RT-PCR [81]. They allow detection of viral RNA in poultry samples, as well as subtyping. In some cases, PCR-positive samples are inoculated into chicken embryos for virus isolation, which is necessary for further antigen and genetic studies [32]. Classical virus isolation in chicken embryos remains the “gold standard”, particularly when a live isolate is required for pathogenicity studies or for full sequencing [82]. Full sequencing allows investigators to trace the origin of the virus, identifying pathogenicity markers and reassortment cases [11]. Operationally, diagnosis follows a two-tier cascade: preliminary screening (ELISA and RT-PCR) is conducted through the Republican Veterinary Laboratory (RVL) branch network, whereas confirmatory testing—RT-qPCR subtyping, sequencing, and virus isolation—is centralized at the National Veterinary Reference Center (NVRC, Astana; Almaty branch) [11,15,66].

As in the world, diagnostic DNA microarrays for detection and subtyping of a diverse set of A/H5N1 influenza viruses are being developed in Kazakhstan [83]. Of particular note is the multiplex microarray developed by Sultankulova et al. (2017), which allows simultaneous detection of, in addition to AIV, Newcastle disease virus (NDV), infectious bronchitis virus (IBV), and avian infectious bursal disease virus (IBDV). Such multiplex diagnostics is of particular importance in conditions where mixed infections may circulate in birds, complicating clinical recognition of the disease. The inclusion of the NP and M2 protein gene zones of the influenza virus in this microarray platform ensures diagnostics at low viral loads [84]. This platform helps to expand monitoring capabilities, as it allows simultaneous detection of several pathogens and subtyping of pathogens by conserved genetic markers. This approach increases the accuracy of diagnostics and makes it more informative for epizootic surveillance. Virus isolation still remains important for obtaining live viruses and conducting antigenic or pathogenicity studies, whereas molecular assays provide rapid and sensitive detection. Among molecular tools, next-generation sequencing (NGS) has already been applied to characterize recent H5N8 and H5N1 outbreaks [11,46], and isothermal amplification methods such as loop-mediated isothermal amplification (LAMP) and recombinase polymerase amplification (RPA) are recognized as promising, rapid, field-adaptable techniques, though not yet widely implemented.

## 6. Vaccination of Poultry Against HPAI in Kazakhstan

While biosecurity, surveillance, and quarantine are the main control measures, vaccination is used as a supplementary strategy to mitigate virus and economic losses, especially in areas with a high risk of HPAI introduction [85,86]. FAO and WOAH have launched the Global Strategy for the Prevention and Control of Pathogenic Avian Influenza (2024–2033) to build resilient poultry production systems, which includes the development of innovative vaccines and surveillance tools to reduce the global disease burden [87,88].

Kazakhstan implements both routine and emergency vaccination of poultry against HPAI, particularly in regions intersecting major migratory bird flyways such as the Kostanay, North Kazakhstan, Akmola, and Pavlodar regions. The use of the domestically developed vaccines contributes to maintaining epizootic stability. In case of disease outbreaks, Kazakhstan relies on inactivated influenza vaccines [71]. In 2007, mass vaccination of poultry began using an inactivated oil-emulsion H5 vaccine developed by the Research Institute for Biological Safety Problems (RIBSP). Recent epidemiological investigations confirm that vaccination is actively applied in Kazakhstan’s high-risk zones, with field monitoring demonstrating seroconversion in vaccinated flocks [15].

According to the WOAH Self-Declaration on HPAI Freedom (2025), the registered monovalent inactivated emulsified H5 vaccine (registration code: RK-VP-1-3413-17) is officially listed in the national register of veterinary drugs and feed additives and used for preventive immunization of chickens, ducks, geese, and turkeys in high-risk regions.

An inactivated vaccine provided a stronger humoral immune response when under a single-dose regimen compared with a baculovirus-based H5 vaccine, indicating that a single-dose inactivated vaccine is more effective for both protection against influenza and maintaining poultry productivity [89,90]. This evidence supports the role of domestic inactivated vaccines as the main tool in national immunization programs.

It has been observed that although vaccination is increasingly used as a control measure and is currently applied in more than 30 countries affected by this infection, experience shows that HPAIV can persist in poultry populations when biosecurity is inadequate and vaccination is used as a sole measure [91]. Thus, the effectiveness of control programs still primarily depends on strict biosecurity, with vaccination serving as an additional measure. In the poultry industry of Kazakhstan, several types of injectable vaccines are currently used to immunize birds against HPAI. These vaccines are registered in the state register of veterinary drugs and feed additives under the Ministry of Agriculture of the Republic of Kazakhstan and within the customs territory of the Eurasian Economic Union, including Russia, Belarus, Kyrgyzstan, and Armenia [91]. It was further demonstrated that commercial vaccines used in Kazakhstan significantly reduced transmission of emergent H5N8 clade 2.3.4.4b strains under field conditions, although long-term success remains dependent on complementary biosecurity measures [91]. In practice, the national regime uses inactivated oil-emulsion H5 vaccines prepared from circulating H5N1 and H5N8 field strains, administered intramuscularly with mineral-oil adjuvants; field studies of an autogenous H5 vaccine have shown protection lasting up to 12 months in chickens [92]. Efficacy is enhanced when vaccine strains are antigenically matched to circulating H5N8 clade 2.3.4.4b viruses, which reduces shedding and onward transmission [91].

## 7. Systematic Support for Surveillance of HPAI in Kazakhstan

The system of regulatory acts of the Republic of Kazakhstan in the field of veterinary and healthcare provides a comprehensive approach to the prevention and control of avian influenza. It includes national regulations and programs as well as regional and local measures aimed at preventing the introduction and spread of infection, protecting public health, and ensuring food security. One of the key regulatory documents is the Law of the Republic of Kazakhstan “On Biological Safety № 122-VII”, which regulates specific aspects of counteracting biological threats, including high-risk infections [93]. In addition to them, ministerial orders are in force, approving sanitary and veterinary rules, as well as National Response Plans and Notifications, in accordance with the requirements of the International Health Regulations (IHR and other regulatory legal acts governing the organization of preventive and anti-epidemic measures in outbreak areas.

In Kazakhstan, surveillance activities for the prevention and response to biological threats, including HPAI, are carried out in cooperation with international structures such as the World Health Organization (WHO) through the Global Influenza Surveillance System (GISRS), the World Organization for Animal Health (WOAH) through the WAHIS information system, the Food and Agriculture Organization of the United Nations (FAO) with data submission to the EMPRES-i system, and the global network of animal influenza experts OFFLU [94].

The FAO reports on the Central Asian region, regularly mentioning Kazakhstan as a country participating in the EMPRES-i system, providing data on outbreaks of HPAI and other diseases. The EMPRES-i system allows international organizations (the FAO and WOAH) to promptly respond to the situation and also provide advisory assistance, clearly formulating the responsibilities of national veterinary services for identifying and reporting outbreaks of influenza in birds. The FAO is developing guidelines for the management of avian influenza outbreaks in poultry farming, focusing on prevention, biosecurity, and interagency coordination [95].

Over the past two decades, with the increasing threat of transboundary epizootics and pandemics, the global risks associated with highly pathogenic avian influenza have led to the development of a number of international strategies aimed at early detection, prevention, and response to zoonotic threats. According to the literature, sustainable containment of avian influenza is possible only with effective coordination between the veterinary and medical spheres within the framework of the One Health concept, and there is a growing need to implement an intersectoral approach reflected in the One Health concept. This concept is based on the recognition of the relationship between human, animal, and environmental health and involves the coordination of efforts of various sectors, including health, agriculture, veterinary medicine, and ecology [76,96,97].

The OFFLU also actively collaborates with the WHO and GISRS within the framework of the One Health concept, combining efforts in monitoring, data sharing, and pandemic preparedness. This collaboration includes joint risk assessments, sharing of virus isolates, and coordination of actions for outbreaks of zoonotic influenza viruses [98].

The WOAH also supports countries in conducting the veterinary system performance assessment (PVS pathway), including interagency collaboration components. Recent research by Mackenzie et al. (2019) and Sikkema et al. (2022) highlights the importance of organizationally structured One Health platforms for joint response to threats [99,100]. The most successful cases have been implemented in the countries of Southeast and Central Asia, where intersectoral centers, joint response teams, and unified information systems are practiced [101]. As one of the appropriate strategies, Lewis et al. noted the interaction between the National Veterinary Reference Centre of Kazakhstan and the UK Animal and Plant Health Agency during the 2020 outbreak in the north–central region of the country, which underscored the subsequent data exchange with the FAO and WOAH. Prior to the dispatch of samples, culling of the affected flocks was carried out, along with the establishment of surveillance zones and vaccination campaigns [102].

For countries with a developed agro-industrial complex, such as the Republic of Kazakhstan, resistance to epizootic threats is directly linked to both biological and economic security. Kazakhstan began exploring the One Health approach to solving the problem of zoonotic diseases in 2018 as part of the National Workshop on Building Linkages (NWBL) between the International Health Regulations (IHR) and the Veterinary Services Performance Assessment Tool (VPS). As a result, a comprehensive long-term national roadmap was developed. The goals of the national roadmap are to establish a sustainable, intersectoral system for the early detection and response to zoonotic infections, develop and implement safety and monitoring standards in livestock production and the food chain, strengthen laboratory diagnostics, train personnel, and establish rapid response teams. Funding for specialist training has been allocated for the 2023–2025 period under the “Healthy Nation” and “Safe Food” national programs, but resource allocation remains uneven across sectors. To overcome jurisdictional and communication barriers, an interdepartmental technical working group and a digital information exchange platform have been established. However, there are still difficulties in aligning priorities, ensuring transparency, and a lack of long-term funding and a legal framework. Based on the roadmap, an action plan was developed to implement the One Health approach, covering not only zoonotic diseases but also food safety and biosecurity, with implementation scheduled for 2025. To ensure regular interaction between ministries, a coordinated government One Health platform was established to improve preparedness for and the response to health challenges at the interface between animals, humans, and the environment. At the same time, efforts were made to stimulate One Health actions, such as the development of a legislative framework for enhanced cooperation and the establishment of an intersectoral research program integrated into the national Healthy Nation project. Responsibility for implementing actions is shared between the health, agricultural, and environmental sectors [103,104,105,106].

The next step was Kazakhstan’s participation in international initiatives: in 2022, with the support of the World Bank, the Central Asian One Health Action Framework was launched, in whose implementation Kazakhstan is actively participating. However, there are currently no public reports showing how the government is distributing resources between the ministries of health, agriculture, and environment within the context of the One Health initiative.

In Kazakhstan in 2024–2025, this work was continued within the framework of a technical working group with the representatives of three ministries (health, veterinary, and ecology). The technical working group held meetings to discuss current issues and proposals on biosafety, interdepartmental coordination, and personnel training.

During practical implementation in Kazakhstan, efforts were made to stimulate actions within the One Health approach, such as developing a legislative framework for expanded cooperation and creating an intersectoral research program integrated into the national Healthy Nation project. Responsibility for implementing these actions was divided among the health, agriculture, and environmental protection sectors. In the Republic of Kazakhstan, this work was continued in 2024–2025 through a technical working group involving representatives from three agencies (health, veterinary, and ecology). The technical working group held meetings to discuss current issues and proposals on biosecurity, interagency coordination, and personnel training. Thus, today, Kazakhstan is developing a sustainable system for training personnel in countering biological threats at the intersection between health, veterinary, and laboratory diagnostics. Particular attention is also being paid to regions located along waterfowl migration routes, which pose an increased risk of virus introduction. As a result of coordinated intersectoral collaboration, outbreaks of HSV in domestic and wild birds were contained and eliminated, preventing transmission to humans.

However, Kazakhstan still lacks a unified interagency platform that would coordinate the efforts of different sectors to ensure effective monitoring, prevention, and control of zoonotic infections.

To strengthen surveillance in practice, we recommend (i) structured monitoring of wild birds at priority wetlands along the CAF with monthly searches for carcasses and quarterly sampling of live birds, with rapid notification to the FAO and the WOAH; (ii) routine whole-genome sequencing of all detections of H5 viruses within the national reference laboratory network, with timely sharing of sequences through international platforms; and (iii) risk-based environmental and serological screening at live-bird markets and smallholder nodes, supported by joint investigation teams from the health and veterinary sectors.

Particular attention is paid to the formation of intersectoral teams, including rapid response teams (RRTs) in the field of healthcare and the training of laboratory service leaders capable of effectively responding to biological threats, including those of transboundary significance [107]. Expert assessments note that, overall, the “One Health” concept in Kazakhstan complies with international standards; however, there remains a bias toward setting objectives and formulating plans rather than presenting concrete results. Most available sources focus on strategic directions and roadmaps, but provide insufficient information on practical steps taken to reduce zoonotic risks on the ground. It is unclear how the effectiveness of measures is assessed; there is a lack of transparent and publicly available data on outbreak dynamics, the coverage of animal outbreaks by preventive measures, and the exchange of epidemiological information between sectors. Furthermore, the adequacy of funding and its distribution among key ministries (health, agriculture, and environment) remains an open question. Comprehensive reports detailing measures by region, population coverage, and specific progress indicators are absent from the public domain. This limited data makes it difficult to objectively assess the extent to which the implementation of the One Health concept has actually strengthened the country’s preparedness for epizootic threats and improved coordination at the intersectoral level.

## 8. Conclusions

Avian influenza remains a pressing threat to both the poultry industry and ecosystems in Kazakhstan. Over the past two decades, the country has repeatedly faced outbreaks of highly pathogenic avian influenza viruses (H5N1 and H5N8), with both poultry and migratory waterfowl species such as swans, geese, and ducks proving to be particularly vulnerable. Despite local monitoring and quarantine efforts, the ongoing transcontinental circulation of these viruses underscores the need for stronger international cooperation. Given Kazakhstan’s important location at the crossroads of bird migration routes, regular virological surveillance and rapid sequencing of pathogens are becoming essential tools in the fight against avian influenza.

Effective prevention of epizootics in poultry in Kazakhstan is not possible without strengthened surveillance of wild birds, especially in the Caspian and northern regions, and without integration of ornithological observations with virological monitoring. Strengthening cooperation between veterinary services, ornithologists, and virologists is also required to ensure a rapid and coordinated response to minimize the consequences of future outbreaks. Continued monitoring and timely response to threats remain an important key element in the fight against avian influenza.

## Figures and Tables

**Table 1:** Summary analysis of highly pathogenic avian influenza viruses (HPAIVs) detected in Kazakhstan and their expected circulation routes in the world.

№	Analyzed Strain	Clade	Phylogeographic Inference	Sources
1	A virus/domestic goose/Pavlodar/1/05 (H5N1)	2.2	Qinghai–Siberian lineage	[19]
2	A/swan/Mangystau/3/2006 (H5N1)	EA-nonGsGD	Probably a reassortant strain; Russian–Far East–Japan	[41]
3	A/flamingo/Mangistau/6570/2015 (H5N1)	2.3.2.1c	Asia–Middle East–Eastern Europe–West Africa lineage	[25]
4	A/chicken/ Akmola/62/21 (H5N8)	2.3.4.4b	Europe–Central Asia–Middle East	[42]
5	A/chicken/North Kazakhstan/184/2020 (H5N8)	2.3.4.4b	Middle East–West Africa lineage	[26]
6	A/Caspian tern/Atyrau/9184/2022(H5N1)	2.3.4.4b	Russian–Caspian Sea	[17]
7	A/Mute swan/Mangystau/9809/2023 (H5N1)	2.3.4.4b	Reassortant strain; Siberia–Egypt–North Africa	[11]
8	A/Cygnus cygnus/Karakol lake/01/2024 (H5N1)	2.3.4.4b	Russia–Caspian Sea	[11,38]
9	A/Mute swan/Karakol Lake/02/2024 (H5N1)	2.3.4.4b	The virus recombined with viruses from ducks in Russia	[11,38]
10	A/mute swan/Mangystau/1-S24R-2/2024 (H5N1)	2.3.4.4b	Egypt–Black Sea–Caspian route	[38]

Abbreviations: HPAIV—highly pathogenic avian influenza virus; EA-nonGsGD—Eurasian lineage non-Goose/Guangdong clade.

**Table 2:** Main periods and places of distribution of H5N1 and H5N8 in Kazakhstan.

Year	Season of Year, Month in Kazakhstan	HPAI Subtypes		Region	Host Category	Species	Sources
		H5N1	H5N8				
2005	Late summer–early autumn	+	-	North Kazakhstan region	Poultry	Geese	[18,43]
					Wild birds	Whooper swans	
2006	Spring: March	+	-	Coast of the Caspian Sea	Wild birds	Swans	[41,44]
2015	Spring: May	+	-	Coast of the Caspian Sea	Wild birds	Flamingoes	[9,25,45],
2020	Autumn: September–November	-	+	Coast of the Caspian Sea North Kazakhstan region	Poultry	Chickens, ducks, and geese	[36,46,47]
2021	Autumn: October	-	+	Qostanay region, North Kazakhstan region, East Kazakhstan region, and Aqmola	Poultry	Chickens, geese, and turkeys	[42]
2022	Summer: June–July	+	-	Coast of the Caspian Sea	Wild birds	Terns and gulls	[17]
2023–2024	Winter: December–January	+	-	Coast of the Caspian Sea	Wild birds	Whooper swans and mute swans	[11,38,48]

Note: “+” indicates presence of outbreaks for the given subtype; “-” indicates absence of outbreaks for the given subtype.

**Table 3:** General list of isolates of highly pathogenic viruses in Kazakhstan and their virological characteristics.

№	Virus Strain, Bird/Place/ Year of the Flu Outbreak	Characteristic Features	Source
1	A/domestic goose/Pavlodar/1/05 (H5N1) (GS/1/05)—domestic geese, northern Kazakhstan, 2005	The presence of a polybasic proteolytic cleavage site of HA; HA, NA, and NS1—increased tropism to mammals, resistance to interferons, and tumor necrosis factor	[19]
2	A/swan/Mangystau/3/2006 (H5N1)—dead swan, southeastern coast of the Caspian Sea, 2006	HA—lacks a polybasic cleavage site; however, according to the WOAH classification *, the strain was considered highly pathogenic, as its intravenous pathogenicity index (IVPI) (N) was 2.34	[54]
3	A/flamingo/Mangistau/6570/2015 (H5N1)—flamingo, Caspian Sea, 2015	The presence of a polybasic proteolytic cleavage site of HA, the PQRERRRKR*GLF motif, and the HPAI marker.	[25]
4	A/chicken/North Kazakhstan/184/2020 (H5N8)—chicken, Northern Kazakhstan 2020	The presence of a polybasic proteolytic cleavage site of HA, the KRRKR/GLF motif (identical to European strains in 2016–2017), and the HPAI marker	[26]
5	A/chicken/ Akmola/62/21 (H5N8)	PLREKRRKR/G cleavage site-marker HPAIV	[42]
	A/wild goose/ Qostanay/83/21 (H5N8)		
	A/domestic goose/Akmola/65/21 (H5N8)		
	A/chicken/ North Kazakhstan/97/21 (H5N8)		
6	A/Caspian tern/Atyrau/9184/2022(H5N1)—terns, north-eastern coast of the Caspian Sea in 2022	The presence of an HA proteolytic cleavage site (PLREKRRKR*GLF); the isolate was classified as HPAIV	[17]
7	A/Mute swan/Mangystau/9809/2023(H5N1)—mute swan, Mangystau 2023	The presence of a polybasic proteolytic cleavage site of the HA—PLREKRRRKR/G marker of HPAIV	[11]
8	A/Cygnus cygnus/Karakol lake/01/2024(H5N1)—whooper Swan Lake Karakol 2024		[11]
9	A/Mute swan/Karakol lake/02/2024(H5N1)—mute swan of Lake Karakol 2024		[11]
10	A/mute swan/Mangystau/1-S24R-2/2024 (H5N1; clade 2.3.4.4b)—mute swan, lake Karakol 2024	The presence of the HA cleavage site—PLREKRRKRGLF marker HPAIV	[38]

* WOAH classifies HPAI by either IVPI ≥ 1.2 in 6-week-old chickens or presence of a polybasic cleavage site.

## Data Availability

All the data presented within this manuscript will be available online.

## Supplementary Materials

The following supporting information can be downloaded at https://www.mdpi.com/article/10.3390/pathogens14111084/s1. Table S1: Detailed gene segment attributions for all isolates.

## Abbreviations

The following abbreviations were used in this manuscript:

EMPRES-i+	FAO’s Global Animal Disease Information System (Emergency Prevention System-Information System Plus)
FAO	Food and Agriculture Organization
GISRS	Global Influenza Surveillance and Response System
IVPI	Intravenous pathogenicity index
HA	Hemagglutinin
HPAI	Highly pathogenic avian influenza
LPAI	Low-pathogenic avian influenza
MOL-PCR	Multiplex oligonucleotide ligation–polymerase chain reaction
NA	Neuraminidase
OFFLU	The WOAH/FAO Network of Expertise on Animal Influenza
RT-PCR	Reverse transcription–polymerase chain reaction
