# Supplementary material for: Epidemiology, Virology, and Control of Highly Pathogenic Avian Influenza in Kazakhstan
Source: Pathogens. 2025 Oct 24;14(11):1084. doi: 10.3390/pathogens14111084 (PMC12655665; doi:10.3390/pathogens14111084)
Supplement: Supplementary file 1 [file pathogens-14-01084-s001.zip › pathogens-3917498-supplementary.pdf]

Supplementary Materials:

Table S1. Detailed gene segment attributions for all isolates

№	Virus Strain, Bird/Place/ Year of the Flu Outbreak	Gene Segments that the Virus Contains; Closest Relative
1	A /domestic goose/Pavlodar/1/05 (H5N1) (GS/1/05) - domestic geese, northern Kazakhstan, 2005	PB2 - A/chicken/Omsk/14/05(H5N1);<br>PB1 - A/Bar-headed Goose/Qinghai/62/05(H5N1);<br>PA - A/turkey/Suzdalka/12/05(H5N1);<br>HA - A/cygnusolor/Croatia/1/2005(H5N1);<br>NP - A/goose/Krasnoozerskoe/627/05(H5N1);<br>NA - A/goose/Suzdalka/10/05(H5N1);<br>M - A/Anas platyrhynchos/ Slovenia/359/06(H5N1);<br>NS - A/goose/Krasnoozerskoe/627/05(H5N1).
2	A/swan/Mangystau/3/2006 (H5N1)- dead swan, southeastern coast of the Caspian Sea, 2006.	PB2 - A/mute swan/Aktau/1460/2006 (H5N1);<br>PB1 - A/mute swan/Aktau/1460/2006 (H5N1);<br>PA - A/mute swan/Aktau/1460/2006 (H5N1);<br>HA[a] - A/mute swan/Aktau/1460/2006 (H5N1);<br>NP - A/greater white-fronted goose/Netherlands/2/2007 (H6N8);<br>NA[a] - A/mute swan/Aktau/1460/2006 (H5N1);<br>M - A/Anas platyrhynchos/Slovenia/359/06 (H5N1);<br>NS[a] - A/mute swan/Aktau/1460/2006 (H5N1);
3	A/flamingo/Mangistau/6570/2015 (H5N1) - flamingo, Caspian Sea, 2015.	PB2 - A/chicken/Nigeria/16VIR5840-34/2015(H5N1);<br>PB1 - A/chicken/Nigeria/16VIR5840-25/2015(H5N1);<br>PA - A/goose/Nigeria/16VIR5840-3/2015(H5N1);<br>HA - A/chicken/Nigeria/16VIR5840-29/2015(H5N1);<br>NP - A/chicken/Nigeria/16VIR5840-56/2015(H5N1);<br>NA - A/chicken/Nigeria/16VIR5840-27/2015(H5N1);<br>M - A/chicken/Nigeria/16VIR5840-68/2015(H5N1);<br>NS - A/goose/Nigeria/16VIR5840-3/2015(H5N1).
4	A/chicken/North Kazakh-stan/184/2020 (H5N8)- chicken, Northern Kazakhstan 2020	PB2 - A/mule_duck/France/20335/2020 (H5N8);<br>PB1 - A/chicken/Kazakhstan/23/2020 (H5N8);<br>PA - A/chicken/Nigeria/VRD21-53B_21VIR2288-5/2021 (H5N8);<br>HA - A/chicken/Kazakhstan/23/2020 (H5N8);<br>NP - A/environment sample/China/TZ001/2021 (H5N8);<br>NA - A/Chlidonias hybrida/Hubei/55/2020 (H5N8);<br>M - A/poultry/Benin/21-A-08-035-O/2021 (H5N1);<br>NS - A/mallard/Shanghai/JDS20876/2020 (H5N8);
5	A/chicken/ Akmola/62/21 (H5N8)	No data
	A/wild goose/ Qostanay/83/21 (H5N8)	
	A/domestic goose/ Akmola/65/21 (H5N8)	
	A/chicken/ North Kazakhstan/97/21 (H5N8)	
6	A/Caspian tern/Atyrau/9184/2022(H5N1) –	The HA, NA and other genes of the virus were closely related to the Russian isolates of clade 2.3.4.4.b from 2022 and other viruses

	terns, north-eastern coast of the Caspian Sea in 2022	from this clade that infected wild birds in Europe, Asia and Africa during the same period.
7	A/Mute swan/Mangystau/9809/2023(H5N1) – mute swan, Mangystau 2023	PB2 - H3N8 virus isolated from ducks (mallards) in the Omsk region of Russia in 2019;<br>PB1 - AIV A/H6N1 isolated from ducks (garganeys) in Egypt in 2022;<br>NP - A/duck/Moscow/6454/2023 (A/H11N9);
8	A/Cygnus cygnus/Karakol lake/01/2024(H5N1) - whooper Swan Lake Karakol 2024	PB2 - H3N8 virus isolated from ducks (mallards) in the Omsk region of Russia in 2019;<br>PB1 - AIV H11N6 isolated from ducks in the Moscow region in 2019;<br>NP - A/duck/Moscow/6131/2022 (A/H3N8);
9	A/Mute swan/Karakol lake/02/2024(H5N1) - mute swan of Lake Karakol 2024	PB2 - H3N8 virus isolated from ducks (mallards) in the Omsk region of Russia in 2019;
10	A/mute swan/Mangystau/1-S24R-2/2024 (H5N1; clade 2.3.4.4b) - mute swan, lake Karakol 2024	PB2 - A/garganey/Egypt/RA20851OP/2022 (H5N1);<br>PB1 - A/Anas platyrhynchos/Belgium/10402_H195386/2017 (H1N1);<br>PA - A/garganey/Egypt/DT20899OP/2022(H5N1);<br>HA - A/garganey/Egypt/DT20899OP/2022(H5N1);<br>NP - A/garganey/Egypt/DT20899OP/2022 (H5N1);<br>NA - A/garganey/Egypt/DT20899OP/2022(H5N1);<br>M - A/duck/Egypt/BA20360C/2022(H5N1);<br>NS - A/chicken/Ehime/TU10-2-13/2022 (H5N1);
